# Preimplantation genetic testing for a family with usher syndrome through targeted sequencing and haplotype analysis

**DOI:** 10.1186/s12920-019-0600-x

**Published:** 2019-11-07

**Authors:** Haining Luo, Chao Chen, Yun Yang, Yinfeng Zhang, Yuan Yuan, Wanyang Wang, Renhua Wu, Zhiyu Peng, Ying Han, Lu Jiang, Ruqiang Yao, Xiaoying An, Weiwei Zhang, Yanqun Le, Jiale Xiang, Na Yi, Hui Huang, Wei Li, Yunshan Zhang, Jun Sun

**Affiliations:** 1 Center for Reproductive Medicine, Tianjin Central Hospital of Gynecology Obstetrics, Tianjin, China; 2 Wuhan BGI Clinical Laboratory Co., Ltd, BGI-Wuhan, BGI-Shenzhen, Wuhan, 430074 China; 3 Tianjin Medical Laboratory, BGI-Tianjin, BGI-Shenzhen, Tianjin, 300308 China; 4 Department of Obstetrics and Gynecology, The Second Affiliated Hospital of Zhengzhou University, Zhengzhou, 450052 China; 5 BGI Genomics, BGI-Shenzhen, Shenzhen, 518083 China; 6 School of Medicine, Nankai University, Tianjin, 300070 China

**Keywords:** Preimplantation genetic testing, Usher syndrome, Targeted capture sequencing

## Abstract

**Background:**

Preimplantation genetic testing for monogenic defects (PGT-M) has been available in clinical practice. This study aimed to validate the applicability of targeted capture sequencing in developing personalized PGT-M assay.

**Methods:**

One couple at risk of transmitting Usher Syndrome to their offspring was recruited to this study. Customized capture probe targeted at *USH2A* gene and 350 kb flanking region were designed for PGT-M. Eleven blastocysts were biopsied and amplified by using multiple displacement amplification (MDA) and capture sequencing. A hidden Markov model (HMM) assisted haplotype analysis was performed to deduce embryo’s genotype by using single nucleotide polymorphisms (SNPs) identified in each sample. The embryo without paternal rare variant was implanted and validated by conventional prenatal or postnatal diagnostic means.

**Results:**

Four embryos were diagnosed as free of father’s rare variant, two were transferred and one achieved a successful pregnancy. The fetal genotype was confirmed by Sanger sequencing of fetal genomic DNA obtained by amniocentesis. The PGT-M and prenatal diagnosis results were further confirmed by the molecular diagnosis of the baby’s genomic DNA sample. The auditory test showed that the hearing was normal.

**Conclusions:**

Targeted capture sequencing is an effective and convenient strategy to develop customized PGT-M assay.

## Background

Usher syndrome is an autosomal recessive disorder that results in hearing loss and progressive retinitis pigmentosa (RP). It is estimated to account for 50% of combined deafness and blindness in adults [1]. The identification of pathogenic variants in high-risk families is helpful for genetic counseling and reproductive management. Once pathogenic variants are identified, preimplantation genetic testing for monogenic defects (PGT-M) can be offered to the affected couple.

PGT-M is a technique used to identify embryos affected by monogenic defects [2]. PGT-M gives prospective couples, which both are carriers of the same recessive monogenic disorders, the opportunity to avoid the occurrence of such diseases. PGT-M was firstly used to avoid the transfer of affected male embryo with X-linked disorders [3]. Allele dropout (ADO) is an inherent defect of single cell polymerase chain reaction (PCR) caused by amplification bias, which is a big threat to diagnostic accuracy [4]. A variety of whole genome amplification (WGA) methods has been developed to address this problem [5]. Multiple displacement amplification (MDA) is becoming a preferred approach for PGT-M due to low error rate and improved genome coverage [2]. However, ADO still occurs in some instances [6]. Several molecular strategies have been developed to determine ADO and avoid the misdiagnosis. Multiplex PCR combined with analysis of linked short tandem repeat (STR) presents significant challenges due to its time-consuming and personalized design [7–9]. SNP array is a fast and universal alternative. SNP array evaluates about 300,000 SNPs throughout the genome [10, 11]. SNP array also enables the testing of whole chromosome aneuploidy and common structural chromosome aberrations. However, the high cost can provide a barrier for clinical application of SNP array [10]. In the past few years, next generation sequencing (NGS) has continuously developed, leading to decreases in the cost and time required for sequencing [12]. NGS-based SNPs haplotyping has been widely applied to PGT-M in clinical practice [13–16].

In this study, we report the development and successful application of targeted capture sequencing and a haplotype analysis-based PGT in an Usher syndrome family, coupled with prenatal testing for fetal aneuploidy and large chromosomal imbalance arrangement, in order to help give birth to a healthy baby.

## Methods

### Patient

The patient was a 27-years-old woman who was reported to have the onset of visual loss and night vision loss 11 years ago. Ophthalmologic examinations showed that she suffered from bilateral RP, and audiometry tests showed that she had a bilateral sensorineural hearing loss. Her father had the onset of visual loss and night vision loss at 30 years and was diagnosed progressive RP 2 years later. Her mother and husband were apparently healthy. The patient had a strong desire to find out the cause of the disease and to have an unaffected child via PGT-M. Approval for this study was obtained from the Institutional Review Board of the BGI. The written informed consents were obtained from all the participants.

### Genetic molecular diagnosis

Molecular genetic analysis using a multiple gene panel (Additional file 1: Table S1) was performed to identify the disease causing-gene for the patient and her parents. Her husband’s sample was also analyzed in parallel as a screening test to check whether he carries any variant in the disease-causing gene. The variants in each family members were confirmed by Sanger sequencing. Detailed information describing experiments and sequencing data analysis is provided in the Additional file 2.

### Preimplantation genetic testing

Detailed information describing in-vitro fertilization (IVF) and PGT-M is provided in the Additional file 2. The SNPs identified in the couple and their parents were used for haplotype construction. The couple’s haplotypes and their associations with variants of interest were constructed through a parents-child trios’ strategy according to Mendel’s law. Maternal haplotype inheritance was deduced with an allele that is homozygous in father and heterozygous in mother. Paternal haplotype inheritance was deduced with an allele that is homozygous in mother and heterozygous in father. We evaluated ADO rate for each embryo sample by using different homozygous alleles in the couple (e.g., mother AA, father BB), and the ADO rate can be calculated as the percentage of allele identified as homozygous in the sample.

An HMM was built based on the allele status in each SNP, and the fetal inheritance haplotypes were deciphered with the Viterbi algorithm as Xu et al. previously described [14]. In order to avoid the influence of ADO in the embryo’s genotype analysis, only heterozygous alleles identified in the embryo sample were used for linkage analysis.

### Prenatal diagnosis

Prenatal molecular diagnosis was performed through amniocentesis at the 20th gestational week. The fetal genotype was confirmed by Sanger sequencing. Aneuploidy and chromosomal imbalanced arrangements larger than 1 Mb were detected with low-coverage whole-genome NGS approach.

### Genetic and auditory examinations of newborns

2 mL EDTA-anticoagulant peripheral blood was collected from the newborn for Sanger sequencing to further confirm the PGT-M and prenatal diagnosis result, and the auditory examination was performed 72 h later after birth by ear acoustic emission analyzer (Maico eroscan).

## Results

### Genetic diagnosis by targeted capture sequencing

Mean sequencing depth of 130-fold per family member was obtained, with an average 91.3% of the region covered with at least 20 reads (Additional file 3: Table S2. Compound heterozygous c.1144-2A > C and c.6752C > A variants in the *USH2A* gene were detected in the patient. The variant c.1144-2A > C was interpreted to be pathogenic referring to the American College of Medical Genetics and Genomics and the Association for Molecular Pathology (ACMG/AMP) guidelines [17] (the evidence: PVS1, PM2, PP5, PP4, PM4). The variant c.6752C > A was interpreted to be pathogenic (the evidence: PVS1, PM2, PP5, PP4, PM3). Descriptions of evidence of pathology for identified variants were provided in Additional file 4: Table S3. The family analysis revealed that the c.1144-2A > C variant was inherited from the patient’s mother who was a carrier of this heterozygous variant, while the variant c.6752C > A (p.Ser2251Ter) was inherited from the patient’s father who was compound heterozygous for the nonsense variant c.6752C > A (p.Ser2251Ter) and a known pathogenic missense variant c.9815C > T (p.Pro3272Leu) [18, 19]. Thus, the pathogenic variant was successfully identified in the family. Both the patient and her father were diagnosed as Usher Syndrome IIA.

**Fig. 1:**
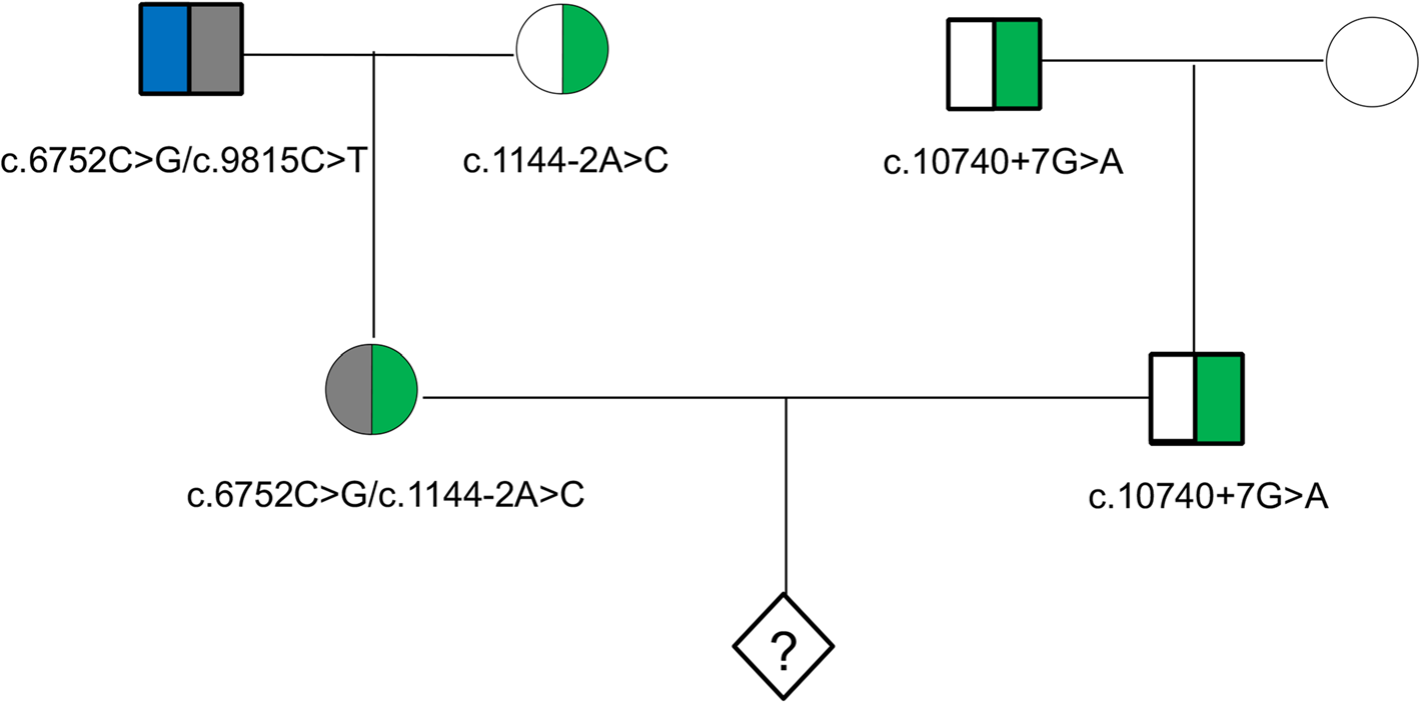
Pedigree of the family. Half-shaded areas indicate carrier state, rhombus indicates embryo

The heterozygous variant c.10740 + 7G > A of *USH2A* was detected in her husband. According to ACMG/AMP 2015 guideline, the c.10740 + 7G > A SNP in *USH2A* gene was classified to uncertain significance (the evidence: PM2, this SNP at extremely low frequency which is below 0.5% in Exome Sequencing Project, 1000 Genomes and ExAC; PP4, patient’s phenotype and her family history are highly specific for Usher Syndrome with a single genetic etiology. Previous studies also supported the correlation between the *USH2A* gene and Usher Syndrome.). It is a variant of unknown significance, but the result predicted by MutationTaster [20] showed that it could form a new donor site. The carrier status of each family member was further validated with Sanger sequencing. (Fig. 1 and Additional file 6: Figure S1).

### Haplotype analysis of *USH2A* gene and 350 kb flanking region in the couple

A mean sequencing depth of 411-fold per family member was obtained (Additional file 5: Table S4). We defined the haplotype linked to c.6752C > A as M-Hap A, while the haplotype linked to c.1144-2A > C as M-Hap B in the patient. For the husband’s haplotype, we defined haplotype linked to c.10740 + 7G > A as F-HapA, and haplotype linked to wildtype allele as F-HapB. We defined a SNP as informative if it was only present in one haplotype. An average of 2883 SNPs were identified in each family member. One thousand one hundred fifteen and One thousand three hundred twenty SNPs were successfully phased in the patient and her husband, respectively. One hundred eighty-four SNPs were identified as heterozygous in the patient but homozygous in her husband and could be used to determine maternal haplotype inheritance, 101 SNPs were M-HapA informative and 83 SNPs were M-HapB informative. Four hundred eighty-three SNPs were identified as homozygous in the patient but heterozygous in her husband and could be used to determine paternal haplotype inheritance, 191 SNPs were P-HapA informative and 292 SNPs were P-HapB informative.

### Genetic diagnosis in clinical PGT cycle

A mean sequencing depth of 189-fold (range: 136–313) was obtained for blood samples and embryo biopsies respectively, with an average 82.1% of the region (range: 68.7–88.2%) covered by at least 30 reads (Additional file 5: Table S4). An average of 41 different homozygous SNPs covered by at least 30 reads in the couple were used for ADO rate calculation. The ADO rate was ranged from 0 to 31.7% in different embryos.

**Fig. 2:**
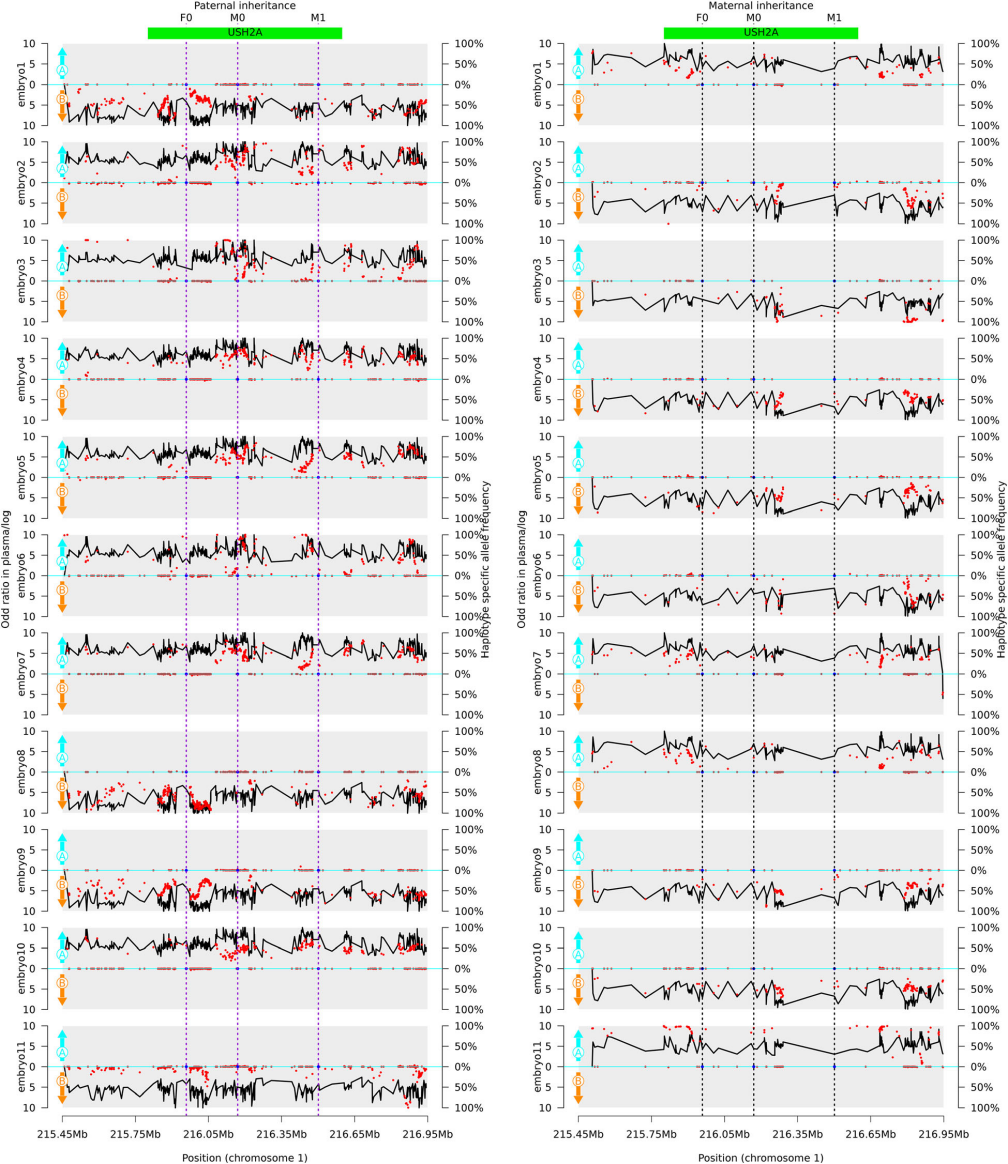
PGT haplotype analysis in embryo 1 to 11. Left, embryo inheritance of paternal haplotype. Right, embryo inheritance of maternal haplotype. The x-axis represents the loci on chromosome 1. The red points represent the allele frequencies of haplotype informative alleles among plasma reads, haplotype A specific allele was drawn above x-axis, and haplotype B specific allele was drawn below x-axis. The black line represents the logarithmic values of the odd ratios of the inherited haplotype. The paternal haplotype A carries c.10740 + 7G > A variant (F0), the paternal haplotype B is wild type. The maternal haplotype A carries p.Ser2251Ter (M0), and the maternal haplotype B carries c.1144-2A > C variant (M1). The vertical dot line indicates the location of target variant

An average of 260 informative heterozygous SNPs (range: 111–379) were identified in embryo biopsy samples, of which an average of 186 SNPs (range: 77–282) were used to deduce paternal inherited haplotype and 73 SNPs (range: 34–97) were used to deduce maternal inherited haplotype. The genotypes of the 11 embryos were all successfully determined using the HMM approach. Four embryos were free of rare paternal *USH2A* variant, embryos 1, 8, 11 were carriers of p.Ser2251Ter variant and embryo 9 was a carrier of c.1144-2A > C. While the other embryos were all compound heterozygous, embryos 2, 3, 4, 5, 6, 10 were compound heterozygotes of c.1144-2A > C and c.10740 + 7G > A, and embryo 7 was a compound heterozygote of p.Ser2251Ter and c.10740 + 7G > A. (Table 1, Fig. 2).

**Table 1:** The haplotype in *USH2A* gene in 11 embryos

Embryo	ADO	Haplotypes in *USH2A* gene	Genotypes in *USH2A* gene	Numbers of Informative SNPs supported each haplotype in each embryo			
				M-Hap A	M-Hap B	F-Hap A	F-Hap B
Embryo 1	0.00%	M-Hap A/F-Hap B	p.Ser2251Ter/N	97	0	0	282
Embryo 2	0.00%	M-Hap B/F-Hap A	c.1144-2A > C/c.10740 + 7G > A	0	66	168	0
Embryo 3	31.71%	M-Hap B/F-Hap A	c.1144-2A > C/c.10740 + 7G > A	0	32	118	0
Embryo 4	0.00%	M-Hap B/F-Hap A	c.1144-2A > C/c.10740 + 7G > A	0	82	183	0
Embryo 5	0.00%	M-Hap B/F-Hap A	c.1144-2A > C/c.10740 + 7G > A	0	82	185	0
Embryo 6	12.20%	M-Hap B/F-Hap A	c.1144-2A > C/c.10740 + 7G > A	0	58	117	0
Embryo 7	0.00%	M-Hap A/F-Hap A	p.Ser2251Ter/c.10740 + 7G > A	94	2	185	0
Embryo 8	4.88%	M-Hap A/ F-Hap B	p.Ser2251Ter/N	95	0	0	276
Embryo 9	0.00%	M-Hap B/F-Hap B	c.1144-2A > C/N	0	83	0	282
Embryo10	0.00%	M-Hap B/F-Hap A	c.1144-2A > C/c.10740 + 7G > A	0	81	181	0
Embryo11	14.63%	M-Hap A/F-Hap B	p.Ser2251Ter/N	34	0	0	77

M-Hap A: p.Ser2251Ter; M-Hap B: c.1144–2 A > C; F-Hap A: c.10740 + 7G > A; F-Hap B: wild type

### Prenatal diagnosis

**Fig. 3:**
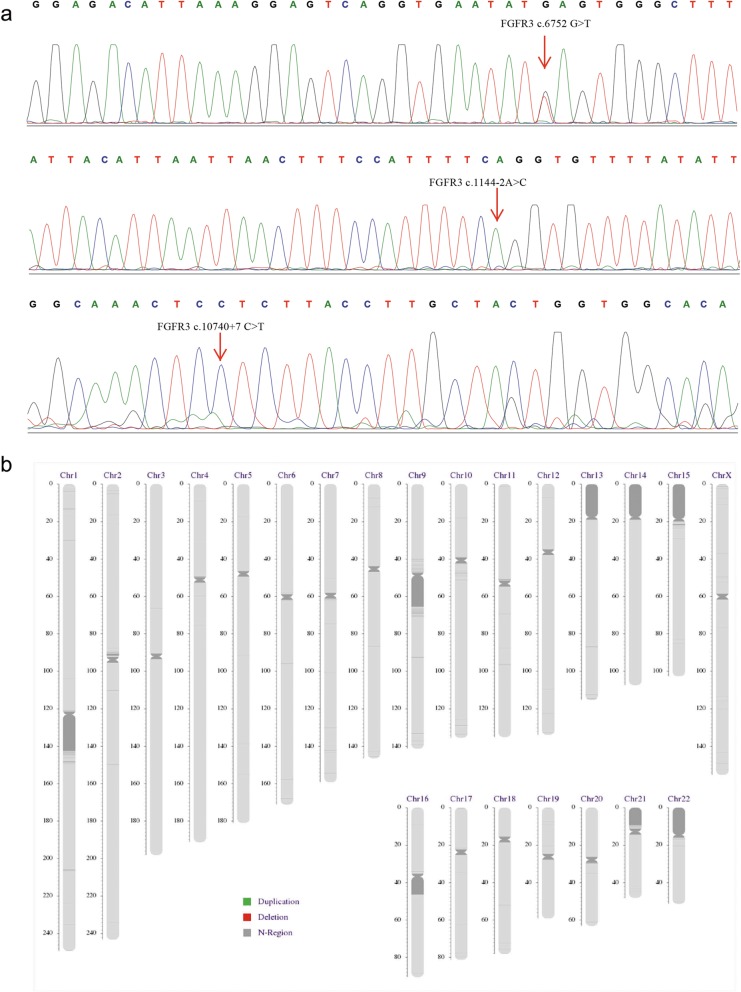
Prenatal diagnosis of amniotic fluid DNA. **a** Sanger sequencing results of amniotic fluid DNA confirmed the PGT results, showing the heterozygous c.6752C > A variant and wild type of c.1144-2A and c.10740 + 7G. And **b** chromosome imbalance anomaly results showed that no CNV larger than 100 kb was identified in the fetus

Embryos 1 was selected for transfer and a successful pregnancy was confirmed by human chorionic gonadotropin (hCG) and ultrasound examination. Sanger sequencing result showed that the fetus was a carrier of p.Ser2251Ter. This confirmed the accuracy of PGT-M (Fig. 3a). The chromosome imbalance anomaly results showed that no copy number variant (CNV) larger than 100 kb was identified in the fetus (Fig. 3b).

### Genetic and auditory examination after birth

A female baby weighting 2850 g was delivered at the 38th gestation week, having apparently normal phenotypes. The PGT-M and prenatal diagnosis result were confirmed with the molecular diagnosis of the baby’s cord blood (Additional file 7: Figure S2a). The chromosome imbalance anomaly results showed that no CNV larger than 100 kb was identified in the baby’s cord blood (Additional file 7: Figure S2b). The auditory examination result was normal.

## Discussion

Usher syndrome is an autosomal recessive disorder and when both parents are carriers for Usher syndrome, each child has a 1 in 4 (25%) chance of inheriting the two changed gene copies, but the risk increase to 1 in 2 if one of the parents is a patient. In this family, affected patients were found in two generations. The clinical symptoms of patient’ father appeared at 30 years when she had been born. Her father was found to carry compound heterozygous variants in the *USH2A* gene. Despite the low carrying rate of pathogenic variant in normal population, the patient still inherited one pathogenic variant from her mother who carried a heterozygous variant in *USH2A* gene. Low-probability events increase pregnant women’s determination to do PGT-M to avoid the Usher Syndrome in the third generation. It has been reported that every person carries an average of 2.8 recessive variants [21]. This also emphasizes the importance of screening the partner of the recessive disease patient to evaluate the risk of conceiving children affected by the same disease. A rare variant (c.10740 + 7G > A) in the *USH2A* gene was detected in her husband. Although the clinical significance of c.10740 + 7G > A is not clear, this variant was predicted to affect the splicing process by MutationTaster, so the pathogenicity can’t be excluded. Since the family already suffers Usher Syndrome for two generations, the patient was unable to tolerate even a little risk for the third generation to avoid the Usher Syndrome. The young couple has a strong desire to receive PGT to prevent the embryo from inheriting c.10740 + 7G > A variant from the husband after enough genetic counseling. Here, we present an NGS- based haplotype linkage PGT-M analysis for an Usher syndrome-affected family.

ADO randomly happens across the genome during MDA, which can result in amplification failure of one of two heterozygous alleles in the embryo and may result in the misdiagnosis of embryo’s genotype. The linkage analysis of genetic markers is an important approach to prevent misdiagnosis that may be caused by ADO in PGT-M. In the past decades, STR was a frequently used genetic marker in PGT, but it is time-consuming and labor-intensive to select appropriate markers for the family of interest and to optimize the experiments. The capture sequencing and linkage analysis of SNPs located nearby the gene of interest provide a convenient and efficient way for PGT-M experiment design. For this couple, 116 informative SNPs (range: 83–292) were identified for each haplotype on average. The estimated ADO rate of each embryo was ranged from 0 to 31.7%. However enough SNPs remained for haplotype analysis of the embryos. Informative SNPs were distributed from upstream of *USH2A* gene to downstream of *USH2A* gene, and this guarantees that any recombination will be identified. The genotype was successfully determined for each embryo. In embryo 7, a recombination event was observed in the maternal allele, but it didn’t influence the genotype deduction of the *USH2A* gene, because the recombination loci were outside the gene region. In this study, we simply captured 1.5 Mb region containing gene region and 350 kb flanking region of *USH2A*. The capture region could be reduced by selecting highly heterozygous SNPs using public databases such as HapMap, dbSNP, 1000 Genome, et al., which can be helpful in reducing the cost of sequencing.

It is noteworthy that invasive prenatal diagnosis is warranted in all cases underwent PGT-M to avoid misdiagnosis [22]. The previous studies have reported that ADO contamination, mosaicism and inappropriate probes or primers were main causes of misdiagnosis via PGT. Chromosomal mosaicism affects up to 50% of early human embryos at the cleavage stage [23]. Chromosomal mosaic rates have been estimated to be as high as approximately 20% at the blastocyst stage biopsy [24]. Biopsy was performed using trophoblast cells rather than inner cell mass which was the true fetal sample. Accordingly, invasive prenatal testing for aneuploidy and chromosomal imbalanced arrangements was carried out to avoid the misdiagnosis due to the chromosomal mosaicism. Couples electing to have PGT do so generally to avoid the chance of having an affected pregnancy. Recently, it has been proved that low-coverage whole-genome NGS is a sensitive and high-resolution method to detect chromosomal aneuploidy and large imbalanced arrangements. It can detect 25% of chromosomal mosaic anomally [25]. Thus, performing an NGS- based invasive prenatal chromosome abnormality detection can provide important genetic information.

## Conclusions

In conclusion, we here present a procedure combining targeted capture sequencing-based PGT-M and invasive prenatal chromosomal anomaly detection in an Usher syndrome-risked family and have obtained a successful outcome. We believe that the targeted capture sequencing is a powerful tool for developing personalized PGT-M, which can be easily extended to other genes. The integrated application of different NGS-based genetic detection methods in various reproductive stages can provide comprehensive information for genetic counseling and clinical decision.

## Supplementary information

**Additional file 1: Table S1.** Genes associated with Usher syndrome and RP.

**Additional file 2.** Supplemental Methods.

**Additional file 3: Table S2.** Statistic of target sequencing for genetic analysis.

**Additional file 4: Table S3.** Descriptions of evidence of pathology for identified variants.

**Additional file 5: Table S4.** Statistic of target sequencing for PGT.

**Additional file 6: Figure S1.** Sanger sequencing result of each family member. a. The result of the patient showed heterozygous of c.6752C > A variant and c.1144-2A > C variant in *USH2A*. b. The result of the patient’s father showed heterozygous of c.6752C > A variant and c.9815C > T variant in *USH2A.* c. The result of the patient’s mother showed heterozygous of c.1144-2A > C variant in *USH2A*. d. The result of the patient’s husband showed heterozygous of c.10740 + 7G > A variant in *USH2A*. e. The result of the patient’s father in law showed heterozygous of c.10740 + 7G > A variant in *USH2A.*

**Additional file 7: Figure S2a.** Sanger sequencing results of the baby’s cord blood. The results showed the heterozygous c.6752C > A variant and wild type of c.1144-2A and c.10740 + 7G in *USH2A* and b. chromosome imbalance anomaly results showed that no CNV larger than 100 kb was identified in the baby’s cord blood.

## Data Availability

The datasets generated and/or analyzed during the current study are not publicly available due to individual privacy or ethical restrictions but are available from the corresponding author on reasonable request.

## Abbreviations

ACMG/AMP: American College of Medical Genetics and Genomics and the Association for Molecular Pathology
ADO: Allele dropout
CNV: Copy number variant
hCG: Human chorionic gonadotropin
HMM: Hidden Markov model
ICSI: Intra-cytoplasmic sperm injection
IVF: In-vitro fertilization
MDA: Multiple displacement amplification
NGS: Next generation sequencing
PCR: Polymerase chain reaction
PGT-M: Preimplantation genetic testing for monogenic defects
RP: Retinitis pigmentosa
SNPs: Single nucleotide polymorphisms
STR: Short tandem repeat
WGA: Whole genome amplification
