# Supplementary material for: Preimplantation genetic testing for a family with usher syndrome through targeted sequencing and haplotype analysis
Source: BMC Med Genomics. 2019 Nov 7;12:157. doi: 10.1186/s12920-019-0600-x (PMC6836415; doi:10.1186/s12920-019-0600-x)
Supplement: Supplementary file 2 — Additional file 2. Supplemental Methods. [file 12920_2019_600_MOESM2_ESM.docx]

**Additional file 2 Supplemental Methods**

**Genetic molecular diagnosis by targeted capture sequencing**

The NimbleGen SeqCap EZ probe was designed to capture exons and 10bp flanking sequences of 67 genes associated with Usher syndrome and retinitis pigmentosa (RP). 5 mL peripheral blood were collected from the patient, her parents, her husband and her parents-in-law, respectively. We extracted genomic DNA (gDNA) from each blood sample and constructed the libraries with 250-bp insert sizes. After hybridization and elution, the libraries were sequenced with 101-bp paired-end reads on Hiseq 2500. The reads were aligned to the human reference genome (hg19) using BWA MEM (0.7.12) with default parameters. The removal of polymerase chain reaction (PCR) duplication reads and verification of mate-pair information were performed with Picard (1.87). Variants were called using GATK (https://software.broadinstitute.org/gatk/) and then annotated and interpreted.

**PCR reaction condition and primers in Sanger sequencing**

The PCR used to amplify mutation of interest was set-up in a final volume of 25 μl, including 50 ng genomic DNA (gDNA), 0.125 μl Ex Taq (Takara), 10 mM dNTP, 0.2 mM of the forward and the reverse primers. Amplification was achieved by a primary step at 95℃ for 15 min, followed by 30 cycles of denaturation at 94 ℃ for 30 seconds, annealing at 59 ℃ for 30 s and extension at 72 ℃ for 1 min, and a final extension at 72 ℃ for 5 min.

| **Variants** | **Forward primer sequence** | **Reverse primer sequence** |
| --- | --- | --- |
| c.1144-2A>C | AGCACCACAATTCCTGGCAAA | TGTTGCTTTTACCACAGGGCT |
| c.6752C>G | GGAGCACTTTTGAGCCACCAA | TGACGAGGACATACCCGAAGG |
| c.10740+7 G>A | ACACCACTTTGAGGAGGGACA | TTTCGGGGAACATCACTGAGC |
| c.9815C>T | ACACTCTAAATCGTTGCTCACA | GCCATGTGTGTATCTGATCTGA |

**In-vitro fertilization (IVF)**

The couple went to Tianjin Central Hospital of Gynecology Obstetrics to undergo an IVF-PGT cycle. The patient underwent an IVF protocol including pituitary down regulation with a gonadotropin hormone releasing agonist. This was then followed by the continued use of the gonadotropin hormone releasing agonist with the addition of a combination of 150 IU of recombinant follicle stimulating hormone (FSH Gonal-F, Merck Serono) and 225 U of human menopausal gonadotropin (HMG, Lizhu China) for a total of 12 days of stimulation. Transvaginal ultrasound monitoring and estradiol measurements were performed to assess follicular maturity with a peak estradiol level of 9556 pg/mL on the last day of stimulation. Human chorionic gonadotropin was administered when there were two lead follicles of 18 mm in average diameter and transvaginal ultrasound guided oocyte retrieval was performed 34 h later. Twenty-four oocytes were recovered and 21 mature oocytes underwent intra-cytoplasmic sperm injection (ICSI).

**Preimplantation genetic testing (PGT)**

21 mature oocytes underwent ICSI and 11 embryos were obtained. Blastocysts biopsy was performed in embryos of grade 3 or higher according to the Gardner’s grading scale on day 6. Approximately five to ten trophectoderm cells were biopsied from each blastocyst. Embryos were vitrified immediately after blastocyst biopsy. Blastocysts biopsy was performed in embryos of grade 3 or higher according to the Gardner’s grading scale on day 6. Approximately five to ten trophectoderm cells were biopsied from each blastocyst. Embryos were vitrified immediately after blastocyst biopsy.

The multiple displacement amplification (MDA) products and gDNA libraries were prepared and captured using 1.5 Mb customized probe covering 350 kb upstream to 350kb downstream of *USH2A* gene. Then the libraries were pair-end sequenced by using Hiseq 2500 with a read length of 101 bp. The sequencing data was analyzed using conventional process provided in the genetic molecular diagnosis section.
