# Supplementary figures and images for: Preimplantation genetic testing for a family with usher syndrome through targeted sequencing and haplotype analysis
Source: BMC Med Genomics. 2019 Nov 7;12:157. doi: 10.1186/s12920-019-0600-x (PMC6836415; doi:10.1186/s12920-019-0600-x)

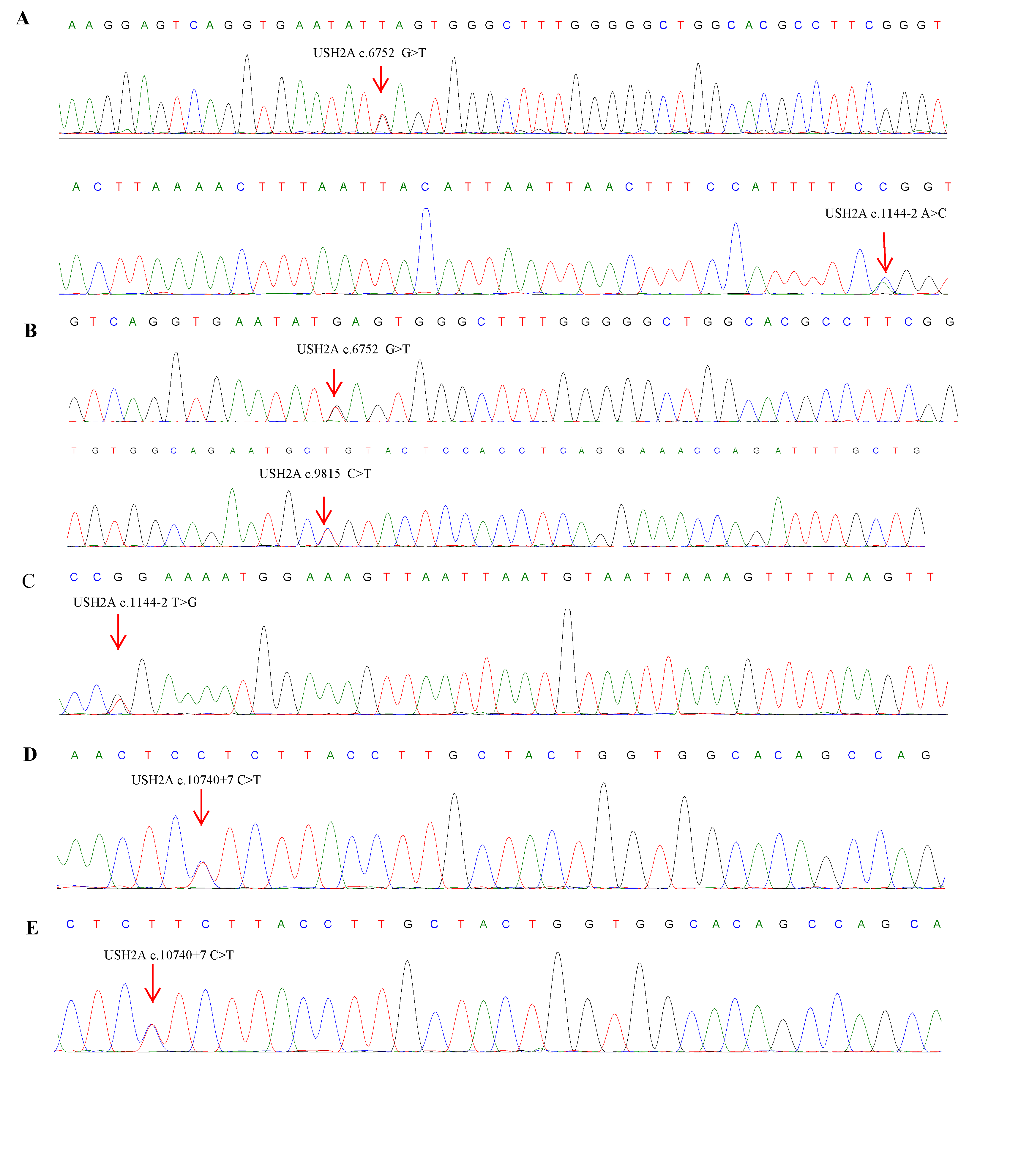

Supplement: Supplementary file 6 — Additional file 6: Figure S1. Sanger sequencing result of each family member. a. The result of the patient showed heterozygous of c.6752C > A variant and c.1144-2A > C variant in USH2A. b. The result of the patient’s father showed heterozygous of c.6752C > A variant and c.9815C > T variant in USH2A. c. The result of the patient’s mother showed heterozygous of c.1144-2A > C variant in USH2A. d. The result of the patient’s husband showed heterozygous of c.10740 + 7G > A variant in USH2A. e. The result of the patient’s father in law showed heterozygous of c.10740 + 7G > A variant in USH2A. [file 12920_2019_600_MOESM6_ESM.tif]
